# How shoulder immobilization influences daily physical activity – an accelerometer based preliminary study

**DOI:** 10.1186/s12891-020-3133-8

**Published:** 2020-02-24

**Authors:** Carolin Rickert, Monika Grabowski, Georg Gosheger, Dominik Schorn, Kristian Nikolaus Schneider, Sebastian Klingebiel, Dennis Liem

**Affiliations:** 1 Department of Orthopaedics and Tumororthopaedics, Albert-Schweitzer-Campus 1 Gebäude A1, University Hospital Muenster, Münster, Germany; 2 Institute of Sports Medicine, University Hospital Muenster, Münster, Germany; 3 Sportopaedicum Berlin, Berlin, Germany

**Keywords:** Shoulder immobilization, Activity of daily living, Sedentary behavior, Rehabilitation

## Abstract

**Background:**

To investigate the influence of shoulder immobilization on daily physical activity.

**Introduction:**

The harmful effect of sedentary behavior does not receive much attention in orthopedic surgery even though immobilization, especially of the lower extremity, has been associated with reduced physical activity. Immobilization of the shoulder is common after reconstructive shoulder surgery and could also potentially lead to reduced physical activity and have a negative effect on a patient’s general health.

**Method:**

Twenty-one healthy volunteers were immobilized in an orthosis (DJO Ultrasling III) for 10 h on two consecutive days. In the following week, activity was measured on the same days without the orthosis. Activity including gait cycles per minute and total gait cycles per day was measured by accelerometer based step count StepWatchTMActivity Monitor. Average age was 26 +/− 3 years. A questionnaire was administered to evaluate subjective activity.

**Results:**

Participants wearing the shoulder orthosis were significantly less active than without immobilization by 2227.5 gait cycles/day (5501.2 with SO, 7728.7 without SO). Also, significantly more time in sedentary behavior occurred (< 400 steps/h) when the shoulder was immobilized. Patients were significantly more active without shoulder orthosis in medium level activities (800–999 steps/h). Differences for low (400–799 steps/h) and high activity levels (> 1000 steps/h) were not statistically significant. Subjective limitations while wearing the orthosis were graded at 2.343 on a scale of 0–4.

**Conclusion:**

Results of this study show that even in young, healthy volunteers immobilization of the shoulder in an orthosis for 2 days leads to significantly reduced activity levels. A negative influence on general health, especially in older patients who are immobilized for up to 6 weeks, can potentially occur. Promoting physical activity during the immobilization period should be part of rehabilitation after injuries/surgery of the shoulder.

**Trial registration:**

Retrospectively registered in DRKS (DRKS00017636).

## Background

Regular physical activity is a clearly proven health resource in prevention and rehabilitation [1] . Physical activity can help prevent cardiovascular disease, diabetes II and obesity as well as numerous other physical and mental disorders [2]. Movement therapy is an important measure for enabling patients to quickly resume their daily professional lives and athletic activities [3]. Physiotherapeutic and other movement therapy measures are of great importance, but daily lifestyle is equally important for faster rehabilitation, recovery and secondary prevention during rehabilitation [4]. Individual lifestyle is not taken into account when designing the rehabilitative therapy concept, however, and it is often not used sufficiently in the rehabilitation, if at all. Hence, sedentary behavior predominates. In the lower extremity, the limitation of physical activity by means of an orthosis is to be expected and is already documented in the literature [5–7] The immobilization of the shoulder joint is a necessary component in the postoperative treatment following shoulder injuries or reconstructive operative interventions in case of shoulder conditions caused by degenerative diseases. Physical activity in this context is thus potentially very significant for elderly patients.

Subjective assessments of patients’ physical activity using questionnaires or self-reporting have been found to be inconsistent. Accelerometry, using devices worn on the body in order to assess activity patterns, has been shown to provide more accurate activity measurements [8]. In contrast to laboratory situations, in which only a snapshot of the patient’s walking ability can be obtained [5], accelerometry provides information about activities during daily life.

But no studies of the activity behavior during wearing of an immobilizing shoulder joint orthosis (SO) have been published in the literature so far. Only in children Maggio et al. documented an activity-related energy expenditure (AEE) due to upper limb cast immobilization in children [9].

The aim of this preliminary study was to evaluate feasibility of this study-protocol and find possible aspects to improve for a larger scale study on actual patients. With regards to the actual content of the study the goal was to measure daily activity in the case of an immobilized shoulder joint in order to derive a transfer to the postoperative therapy program following shoulder operations and to expand possible recommendations for rehabilitation therapy.

## Method

### Participants

Twenty-one volunteers recruited from fellow students (8 men and 13 women between the ages of 20 and 30) have been included in this cohort study (see Table 1).

**Table 1:** Demographic data

	N	Age [year]	Weight [kg]	Height [m]	BMI [kg/m^2^]
Total	21	26 ± 3	71.19 ± 11.0	174.95 ± 7.12	23.23 ± 2.62
Female	13	25 ± 3	65.85 ± 7.89	171.31 ± 6.19	22.45 ± 2.390
Male	8	27 ± 4	79. 88 ± 9.99	180.88 ± 3.8	24.51 ± 2.58

Inclusion criteria:

- ▪Between 18 and 35 years of age signed an informed consent form

Exclusion criteria:

- ▪Acute or chronic diseases of the cardiovascular system or the passive / active musculoskeletal system

### Immobilization of the shoulder joint

The shoulder joint was immobilized by means of the DONJOY® GOBAL shoulder joint orthosis (DJO Ultrasling III). An abduction of 10°–15° is carried out in the lower arm sling with a connected body pillow.

### Objective measurement of daily activity using step counter

Daily activity was measured using an accelerometer-based step counter (*SAM*: *StepWatch™ 3.0 Activity Monitor*) at two different measurement times. Measurement 1 was conducted with the shoulder joint orthosis for 10 h on two consecutive weekdays. Measurement 2 was conducted the following week on the same days in the same period, with comparable daily activities for 10 h without the shoulder joint orthosis.

Using a belt, the device is attached to the right leg laterally and above the ankle, or alternatively on the left leg above the medial ankle.

Horizontal and vertical directional changes are measured by means of integrated acceleration sensors. The internal storage capacity of 32 KB allows a continuous recording of 60 days, while the running time of the internal battery is 5–7 years [10] when used continuously.

*StepWatch™ 3.4 Analysis Software* was used to program the device before the measurement and to evaluate the raw data of the gait cycles after the measurement. The monitor has been validated in several studies and has been reported to have a 99% accuracy rate in detecting gait cycles per time interval [11, 12].

Gait cycles per minute and total gait cycles per day were documented. Based on the gait cycles / day, four activity categories can be established following Tudor-Locke and Craig [13, 14] (see Table 2).

**Table 2:** Activity categories of adults according to Tudor-Locke and Craig

	Activity category	gaitcycles/day	gaitcycles/hour
1	**Sedentary behavior**		
		< 5.000	< 400
	Basal activity	< 2.500	
	Limited activity	2500–4.999	
2	**Light activity**		
	low active	5.000–7.499	400–599
	somewhat active	7.500–9.999	600–799
3	**Moderate activity**	10.000–12.499	800–999
4	**Virgous activity**	≥12.500	≥1000
	highly active		

### Questionnaire for the assessment of subjective daily activity

An individual questionnaire was administered to provide documentation of the subjective feeling of the test person during the measurement with an accelerometer and shoulder orthosis as well as without an orthosis (See Additional files 1 and 2).

### Questionnaire for the subjective assessment of restriction by the shoulder orthosis

Documentation was also carried out concerning the subjective restriction caused by the shoulder orthosis. The questionnaire items were assessed on the basis of a four-step scale, where 1 refers to the most strongly perceived restriction and 4 being the least restrictive (See Additional file 2).

### Statistics

The statistical evaluation was performed by Microsoft Office 2016 (Microsoft Corporation, Redmond, WA), Microsoft Excel 2016 (Microsoft Corporation, Redmond, WA), SPSS 23 for Mac (IBM Corporation, NY) and StepWatch™ 3.4 Analysis Software.

After examination of the application requirements by means of the Kolmogorov-Smirnov test, the following analytical test procedures were carried out:

- ▪ Kolmogorov-Smirnov test (for age, gender and BMI)
- ▪ *t* test for dependent samples (total gait cycles with/ without SO, activity levels with/ without SO)
- ▪ Wilcoxon test for non-parametric distributions (activity level with/without SO)
- ▪ Correlation test of Spearman / Pearson (activity level and total gait cycles)
- ▪ Reliability analysis according to Cronbach’s alpha

The error probability (p) is set to 5% for all tests.

## Results

### Participants

The study included a sample of 21 subjects (*N* = 21), of whom 38.1% were male (*N* = 8) and 61.9% were female (*N* = 13). One subject wore the accelerometer incorrectly (data lost was on all measurement sessions of one person), meaning only the data from 20 subjects could be evaluated with regard to the total cycle number. The average age was 26.1 ± 3.6, and the average BMI was 23.23 ± 2.6.

### Compliance and adverse events

All participants were compliant to wearing the shoulder orthoses. However, one patient had to be excluded due to incorrect wearing of the accelerometer.

On average, the patients stated moderate restriction of the shoulder orthoses (2.343 points). No adverse events occurred.

### Total gate cycles (totcyc) with and without the shoulder orthosis

**Fig. 1:**
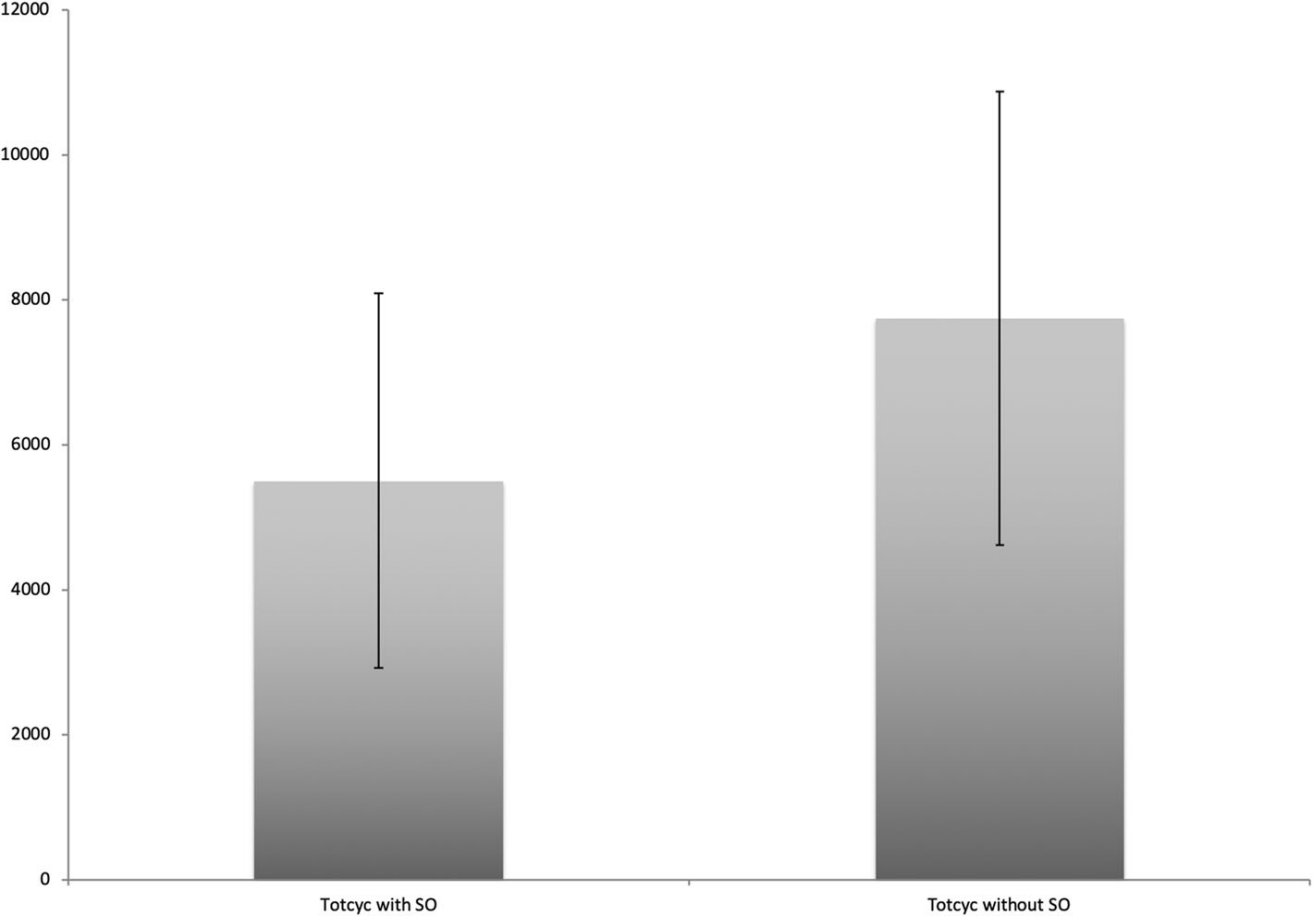
Total gait cycles (Totcyc) with and without the shoulder orthosis (SO)

In the measurement period with the shoulder orthosis, 5501.2 ± 2580.94 totcyc is significantly less than in the second measurement period without the shoulder orthosis (7728.7 ± 3121.76 totcyc) (*p* < 0.05) (see Fig. 1).

**Fig. 2:**
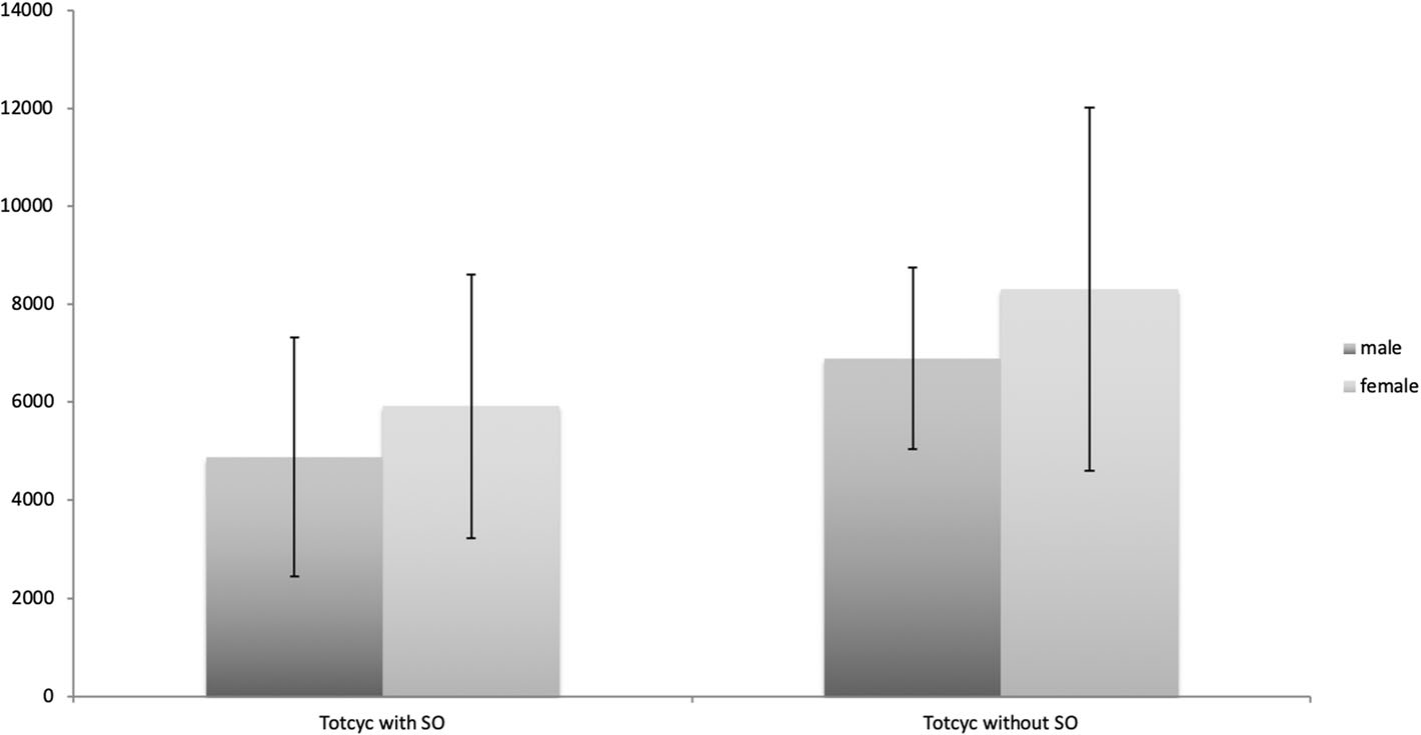
Graphical representation of the totcyc with and without shoulder orthosis differentiated by gender

Between gender and the totcyc in daily life, there is a positive weak linear relationship of r = 0.159 with the shoulder orthosis and r = 0.142 without the shoulder orthosis. The difference between gender and the step number with or without the SO is statistically not significant (see Fig. 2).

**Fig. 3:**
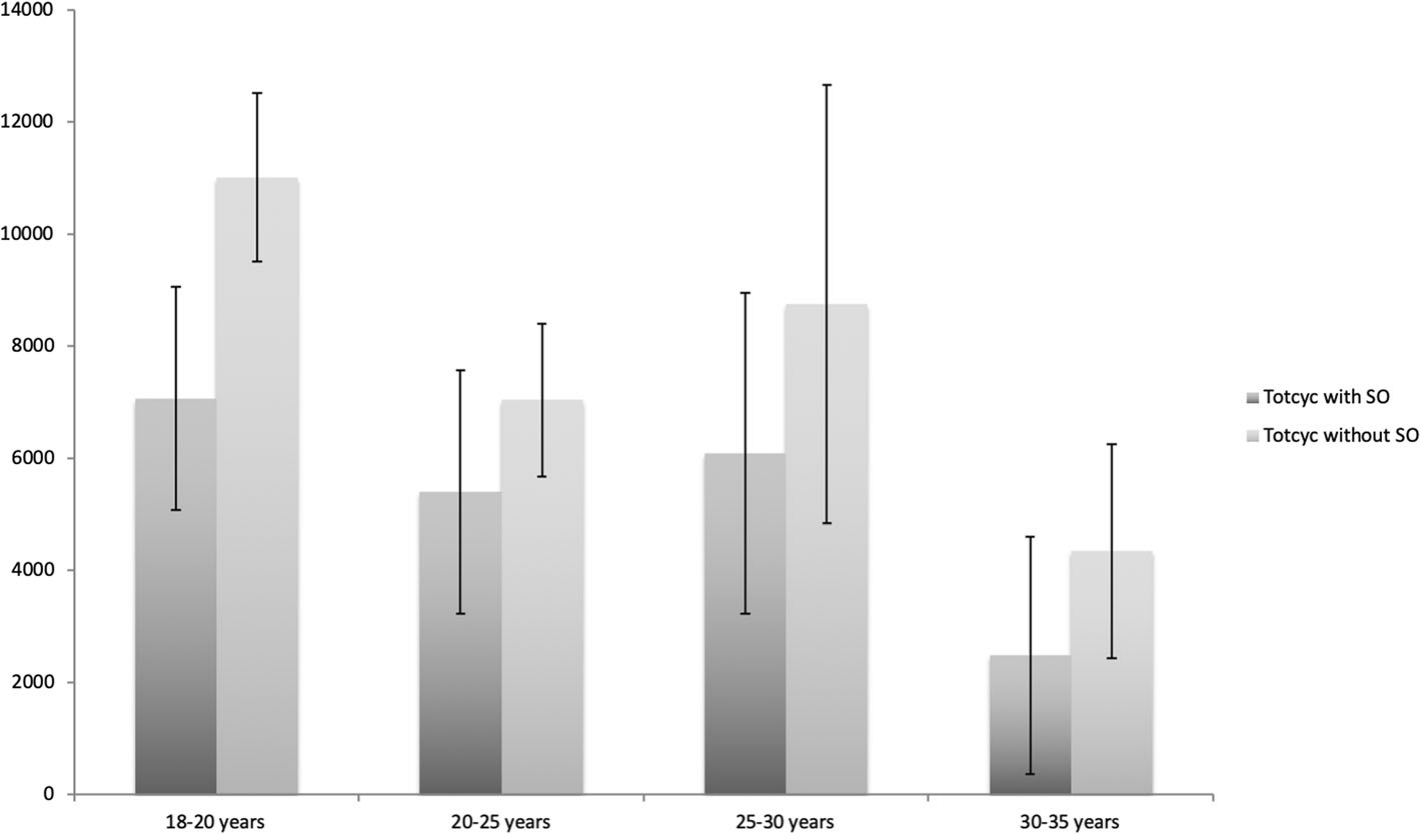
Graphical representation of the totcyc with and without shoulder orthosis differentiated by age categories

According to the rank correlation analysis of Spearman-Rho, there is a negative linear relationship between age and the total cycles with and without the SO, which is interpreted as a weak correlation with r = − 0.160 (with the SO) and r = − 0.201 (without the SO), which is not significant (*p* > 0.05) (see Fig. 3).

### Subjective assessment of daily activity

The subjective activity was not estimated consistently by the participants with respect to the activity categories. Subjects who assessed themselves as *somewhat active* made on average even more steps with the SO (5964.09 steps / day) than the subjects with a self-assessment of an *active* lifestyle (4845.83 steps per day). The two subjects who assessed themselves as *low active* have a *sedentary* lifestyle according to the activity categories of the modified table according to Tudor-Locke. The 11 volunteers who assessed themselves as *somewhat active* assessed themselves correctly on average. The subjects who assessed themselves as *active* walked without an orthosis of the shoulder, according to the activity categories in Table 2, an average of 8201.50 totcyc, which falls under the category of a *somewhat active* lifestyle. All subjects who assessed themselves as *active* fall under the category of a *somewhat or even low active* lifestyle.

### Activity level with and without the shoulder orthosis (SO)

Regarding the activity level there are significant differences to be found between the first and second measurements only in the “inactive” and “medium active” levels.

Regarding the health recommendations about the steps to be taken per day, the subjects averaging 5501.2 steps on the days with shoulder orthosis fall under the activity category of light activity, which corresponds to a low active lifestyle. By comparison, the activity of the subjects on the days without shoulder orthosis is significantly higher. With an average of 7728.7 steps per day, the subjects fall under the category of “somewhat active.”

## Discussion

The aim of this study was to evaluate the effect of wearing a shoulder orthosis on the daily activity of healthy volunteers in order to draw conclusions in a next step on the patient’s activity after shoulder surgery.

In their study, Coleman et al. documented 53 patients with an average age of 75.4 years old with an immobilized shoulder which negatively affected balance and increased the risk of falling [15]. Based on the results of their study, the authors recommend early balance training and fall prophylaxis during rehabilitation after shoulder surgery. In a further study, Hatta et al. investigated the influence of the arm position during the immobilization by means of shoulder orthosis in 20 healthy volunteers, considering comfort and the associated effects on daily activity [16]. It is shown here that an abduction of the arm up to 60° does not have any effect on the restrictions on ADLs, but there is also a clear subjective discomfort with corresponding restrictions on daily activities.

In the present study, wearing of the shoulder orthosis affected the daily physical activity by significantly lowering the step cycles (*p* = 0.000). The inconvenience caused by wearing the shoulder orthosis was most strongly affirmed (2.343). The item of motivation has been confirmed with 1.95 as the second most significant after the item of the unpleasant feeling with 1.80. This suggests that the subjects’ psychological mood was influenced by wearing the shoulder orthosis and thus it minimized their daily physical activity.

In terms of the activity level, the subjects walked 5501.2 steps / day with the SO, which corresponds to a *low active* lifestyle. Effects harmful to health can thus be exacerbated due to low physical activity caused by wearing the shoulder orthosis. In comparison to the measurement without a shoulder orthosis, the subjects walked an average of 7728.7 steps / day, which is in the range of an *active* lifestyle. Wearing a shoulder orthosis thus significantly (*p* = 0.000) contributes to the activity level decreasing from *moderate activity* to the harmful level of *light activity*.

The subjects without the SO figured significantly (*p* = 0.003) less in the inactive activity range (68.67%) than with the SO (62.85%) (see Fig. 2). This suggests that physical activity increased during the day because of less sedentary behavior.

As expected, the subjects assessed wearing the SO as “obstructive.”

The overall activity of healthy subjects is significantly restricted in general. Thus, a restriction of the activity of older patients with an immobilized shoulder is to be expected. Therefore interventions for gaining more activity like special self-exercise programs or ergometer training would be useful in the rehabilitation after shoulder surgery.

### Limitations

This study is a prelimanary study which in part is to establish feasibility of the testing protocol. Further studies with older patients who have undergone shoulder surgery with 3–6 weeks of immobilization postoperatively are being planned for investigating the concerning population.

Due to the small sample (*N* = 20), conclusions cannot be generalized. Since this study is a pilot study, further studies are necessary to be able to draw conclusions about the general population.

Validity of the self-made questionnaires are not given.

There is no comparability with other studies since there are no comparable studies in the literature.

## Conclusions

The overall activity of healthy active subjects is adversely affected to a significant extent by wearing a shoulder orthosis, and sedentary behavior is increasingly observed. In addition to the objectively measured restriction, the results of the questionnaire confirm a subjectively perceived restriction.

A negative impact of wearing a shoulder orthosis, especially in elderly patients, who often wear an SO after shoulder operations, can therefore be expected. This should be an incentive to carry out activity-enhancing measures in daily life in the rehabilitation of shoulder injuries / operations.

## Supplementary information

**Additional file 1.** Questionnaire for the assessment of subjective daily activity.

**Additional file 2.** Questionnaire for the subjective assessment of restriction by the shoulder orthosis and daily activity.

## Data Availability

The datasets used and analysed during the current study are available from the corresponding author on reasonable request.

## Abbreviations

ADL: Activity of daily living
BMI: Body mass index
SO: Shoulder joint orthosis
Totcyc: Total gate cycles
