# Supplementary material for: How shoulder immobilization influences daily physical activity – an accelerometer based preliminary study
Source: BMC Musculoskelet Disord. 2020 Feb 24;21:126. doi: 10.1186/s12891-020-3133-8 (PMC7041289; doi:10.1186/s12891-020-3133-8)
Supplement: Supplementary file 1 — Additional file 1. Questionnaire for the assessment of subjective daily activity. [file 12891_2020_3133_MOESM1_ESM.docx]

**Questionnaire**

How Shoulder Immobilization Influences Daily Physical Activity – An Accelerometer Based Pilot Study

1. **Individual-rated data**
2. Gender
3. Age
4. Height
5. Weight
6. BMI
7. **Physical activity**
8. Are you employed?
9. If so, what kind of activity includes your job?

- Sedentary activity (office, student)
- Moderate activity (manufacturer, housewife, caretaker)
- Intensive activity (mailman, builder)

1. How would you rank your physical activity?

- Very little active
- Little active
- Moderate active
- Active
- Very active
