# Supplementary material for: How shoulder immobilization influences daily physical activity – an accelerometer based preliminary study
Source: BMC Musculoskelet Disord. 2020 Feb 24;21:126. doi: 10.1186/s12891-020-3133-8 (PMC7041289; doi:10.1186/s12891-020-3133-8)
Supplement: Supplementary file 2 — Additional file 2. Questionnaire for the subjective assessment of restriction by the shoulder orthosis and daily activity. [file 12891_2020_3133_MOESM2_ESM.docx]

**Questionnaire**

How Shoulder Immobilization Influences Daily Physical Activity – An Accelerometer Based Pilot Study

**Your physical activity in the last two days**

|  | Strongly disagree | Disagree | Agree | Strongly agree |
| --- | --- | --- | --- | --- |
| Did the last 48 hours run common for you, so they are comparable with other days? |  |  |  |  |
| Is your physical activity in the last 48 hours comparable to what you are doing usually? |  |  |  |  |
| Did the orthosis handicap activities not concerning your arm? |  |  |  |  |
| Did your physical activity differ from a usual day without orthosis? |  |  |  |  |
| Was your physical activity less than usual? |  |  |  |  |
| Did you feel motivated to do physical activity the last 48 hours? |  |  |  |  |
| Did your mood change concerning physical activity? |  |  |  |  |
| Do you feel tired and inactive? |  |  |  |  |
| Were you able to handle your everyday job? |  |  |  |  |
